# The Association between Belgian Older Adults’ Physical Functioning and Physical Activity: What Is the Moderating Role of the Physical Environment?

**DOI:** 10.1371/journal.pone.0148398

**Published:** 2016-02-12

**Authors:** Veerle Van Holle, Jelle Van Cauwenberg, Freja Gheysen, Delfien Van Dyck, Benedicte Deforche, Nico Van de Weghe, Ilse De Bourdeaudhuij

**Affiliations:** 1 Department of Movement and Sport Sciences, Ghent University, Ghent, Belgium; 2 Department of Public Health, Ghent University, Ghent, Belgium; 3 Department of Human Biometry and Biomechanics, Vrije Universiteit Brussel, Brussels, Belgium; 4 Department of Geography, Ghent University, Ghent, Belgium; 5 Research Foundation Flanders (FWO), Brussels, Belgium; Universidad Europea de Madrid, SPAIN

## Abstract

**Background:**

Better physical functioning in the elderly may be associated with higher physical activity levels. Since older adults spend a substantial part of the day in their residential neighborhood, the neighborhood physical environment may moderate associations between functioning and older adults’ physical activity. The present study investigated the moderating role of the objective and perceived physical environment on associations between Belgian older adults’ physical functioning and transport walking, recreational walking, and moderate-to-vigorous physical activity.

**Methods:**

Data from 438 older adults were included. Objective physical functioning was assessed using the Short Physical Performance Battery. Potential moderators included objective neighborhood walkability and perceptions of land use mix diversity, access to recreational facilities, access to services, street connectivity, physical barriers for walking, aesthetics, crime-related safety, traffic speeding-related safety, and walking infrastructure. Transport and recreational walking were self-reported, moderate-to-vigorous physical activity was assessed through accelerometers. Multi-level regression analyses were conducted using MLwiN to examine two-way interactions between functioning and the environment on both walking outcomes. Based on a previous study where environment x neighborhood income associations were found for Belgian older adults’ moderate-to-vigorous physical activity, three-way functioning x environment x income interactions were examined for moderate-to-vigorous physical activity.

**Results:**

Objectively-measured walkability moderated the association between functioning and transport walking; this positive association was only present in high-walkable neighborhoods. Moreover, a three-way interaction was observed for moderate-to-vigorous physical activity. Only in high-income, high-walkable neighborhoods, there was a positive association between functioning and moderate-to-vigorous physical activity. No functioning x walkability interactions were observed for recreational walking, and none of the perceived environmental variables moderated the positive association between physical functioning and the physical activity outcomes.

**Conclusions:**

For older adults with better physical functioning, living in a high-walkable neighborhood could be beneficial to engage in more transport walking. Living in high-income, high-walkable neighborhoods and having better functioning might also be beneficial for more engagement in moderate-to-vigorous physical activity. This might suggest a protective role of neighborhood walkability for preventing declining physical functioning and consequently decreasing physical activity levels in older adults. However, given the cross-sectional design of the present study, this suggestion needs to be confirmed through longitudinal assessment investigating over-time changes in the observed associations.

## Introduction

It is expected that the worldwide proportion of older adults (≥65y) will increase substantially within the forthcoming years [1]. Since physical constraints and functional limitations typically increase with age [2], aging may also affect the health care sector through a rise in the number of institutionalized individuals. At older age, maintaining an active lifestyle through regular engagement in moderate-to-vigorous physical activity (MVPA) may decelerate age-related declines in physical functioning [3–7], so that in the long term, older adults can live independently for a longer time.

Apart from the expected positive effects of regular physical activity (PA) on older adults’ physical functioning, the association between these health-related variables operates in both directions: physical functioning may also be positively associated with older adults’ PA participation [8,9] or, stated otherwise, poorer physical functioning may be related to higher inactivity in the elderly. Age-related decreases in physical functioning are characterized by a musculoskeletal [10,11] and neural [12] degeneration, which induce difficulties in moving around and being physically active. Besides, older adults with lower physical functioning often experience fear of falling [13], an important age-related issue that may jeopardize PA participation as well [14–16]. Older adults with poor physical functioning may thus be at a higher risk of physical inactivity [17] and a downward spiral pattern may exist between decreasing PA and declines in physical functioning [18]. Because of age-related declines in physical functioning levels, older adults can be considered a population at risk for adverse health outcomes due to physical inactivity. Hence, older adults are an important target group for interventions aimed at increasing PA levels.

Although there seems to be a direct association between physical functioning and older adults’ PA, it is likely that this relationship is more complex and may be influenced by modifiable factors, with the neighborhood physical environment being one potential important factor [19–21]. As a result of retirement, older adults are more likely to spend a great amount of time in the home and neighborhood environment towards spending most of the time in the home environment [22]. Hence, community-dwelling older adults are more likely to be affected by their neighborhood environment [23] and could be more susceptible to (changes in) aspects of the neighborhood physical environment because of a higher exposure. Previous research has shown some evidence on the importance of the objective and perceived physical environment in relation to older adults’ domain-specific and overall PA [24–30] and this association may be moderated by neighborhood income [30]. Especially older adults with lower physical functioning may be more strictly bound to their residential neighborhood because of decreased mobility. In addition, press-competence models [31] posit that when individuals’ capacities decrease, they become more sensitive to environmental pressure (e.g., barriers such as large distances to destinations). Hence, the more functionally limited may feel more vulnerable to the physical environment because they experience more environmental barriers to engage in PA, and the importance of an accessible physical environment (e.g. having shops at short distance) may become more important with regard to their PA participation [32,33]. Taking this into account, it could be hypothesized that the positive association between older adults’ physical functioning and PA levels may be stronger in environments that are less activity-supportive and attenuated in activity-friendly environments. That is, an environment that is more supportive of PA (e.g., has better infrastructure for walking) is likely to boost PA participation especially in older adults with poor physical functioning. On the other hand, the functioning-PA association is expected to be less pronounced in more activity-friendly environments, because the activity-friendliness of the environment may possibly counter possible lower PA participation associated with poorer physical functioning.

To date, it is still unclear which environmental factors are most important or activity-friendly [34] and to our knowledge, little research investigated possible interactions between the physical environment and older adults’ physical functioning in relation to PA. Moreover, the few existing functioning x environment interaction studies in older adults observed results that are contrasting with the hypothesis previously described [29,35]. King et al. [29] observed that the positive association between better functioning and older adults’ transport-related PA was stronger when these older adults were living in a high-walkable (i.e. more activity-friendly) environment. Furthermore, Satariano et al. [35] examined the interaction between the objective environment and functioning to predict walking levels in the elderly. Although the authors considered older adults’ physical functioning (and not the physical environment) as a moderator, the direction of the interaction was similar to the one observed in the study of King et al. [29]. Specifically, Satariano et al. [35] found a positive association between objectively-measured street connectivity and higher odds of walking at least 150 minutes/week only in older adults with better physical functioning, whereas no association was observed in those with poorer functioning levels. So, for interactions between functioning and the *objective* physical environment, synergistic effects between higher levels of functioning and more activity-friendly physical environments were observed in these US studies. In contrast, findings on the interaction between physical functioning and the *perceived* physical environment were less straightforward. In the Satariano et al. [35] study, perceived crime was associated with lower levels of older adults’ PA only for those older adults with poorer physical functioning, but no other perceived environmental variables interacted with older adults’ functional status in explaining differences in PA levels. It should be noted, however, that both of the above-described moderation studies were conducted in the US, and the observed associations may differ from those in other geographic areas, because of physical environmental [36] or socio-cultural variation. Hence, it is important to examine associations between physical functioning levels and older adults’ PA levels, and to identify possible moderating effects of the physical environment in countries other than the US.

The aim of the present study was to investigate whether the objective and perceived physical environment moderate the relationship between Belgian older adults’ physical functioning and their PA levels. We hypothesized a positive association between functioning and older adults’ PA, which was expected to be more pronounced when objective or perceived environmental factors would be less favorable (i.e. low-walkable and poorer environmental perceptions). Objectively-measured MVPA, as well as domain-specific walking (self-reported) were used as outcome measures in the present study. In addition, for MVPA, three-way interactions between functioning, the environment and neighborhood income were assessed, since neighborhood income was observed as a significant moderator on the environment-MVPA association in a previous study among Belgian older adults [30].

## Methods

### Sampling and procedures

Cross-sectional data from the Belgian Environmental Physical Activity Study in Seniors (BEPAS Seniors) were used in the present study. Data collection was performed between October 2010 and September 2012 among community-dwelling older adults (≥65y) living in 20 neighborhoods located in Ghent and its suburbs. A detailed description of neighborhood selection and participant recruitment can be retrieved elsewhere [30]. Briefly, neighborhoods were stratified on Geographic Information System (GIS)-based walkability (high vs. low) and matched on neighborhood annual household income (high vs. low). This matching resulted in four neighborhood strata: high walkability/high income, high walkability/low income, low walkability/high income, and low walkability/low income. Stratified sampling (based on gender and age [<75y vs. 75y and older]) was applied to recruit older adults in each neighborhood. Potential participants were sent an informative recruitment letter, explaining the purpose of the study and announcing the visit of a trained interviewer during the subsequent two weeks. Criteria for inclusion in the study were: being able to understand and speak Dutch, living independently (non-institutionalized), and being able to walk a couple of hundred meters without severe difficulties. Response rate was 40.3% (508/1135 eligible participants found at home). Responders were visited twice by the trained interviewer, with a mean interval of nine days in between two visits. During the first visit, respondents gave written consent for participation in the study and for their clinical records to be used in this study. After giving written consent, respondents participated in a face-to-face interview assessing self-reported walking in the preceding week and perceived neighborhood environmental factors. Furthermore, the trained interviewer instructed participants how to wear an ActiGraph GT3X(+) accelerometer for measuring objective moderate-to-vigorous physical activity (MVPA). The second home visit consisted of collecting the accelerometer, assessing information on socio-demographics by means of a face-to-face interview and conducting a short test battery for assessing lower-extremity physical functioning. The study protocol was approved by the Ethics Committee of the Ghent University Hospital (registration number B670201423000).

### Measures

#### Socio-demographics

Participants self-reported their age, current living situation (responses were dichotomized into ‘having a partner’ and ‘not having a partner’), educational attainment (responses were dichotomized into ‘tertiary education’ and ‘non-tertiary education’), and former occupational status (responses were categorized into ‘household’, ‘blue collar’, and ‘white collar’).

#### Physical functioning score

Physical functioning was assessed using the Short Physical Performance Battery (SPPB) [37]. The SPPB outcome measure was found to be a predictor of mortality and nursing home admission in initially non-disabled older adults [37] and can be considered an appropriate tool to assess physical functioning in older adults. The test battery includes three tests of lower-extremity functioning: five repeated chair stands, a balance test (side-by-side; semi-tandem; tandem position) and a three meters walking test. For each of the three tests, time to complete the task was recorded and based on the scoring protocol by Guralnik et al. [37] an ordinal score (range 0–4) was awarded. Consequently, a summary score was computed by summing the three ordinal scores. This summary score ranged from 0 to 12, with higher scores reflecting better physical functioning.

#### Objective potential moderator: GIS-based neighborhood walkability

The neighborhood-level walkability index applied for the present study was calculated using GIS data on residential density, street connectivity and land use mix diversity of Ghent, as described in a study by Van Dyck et al. [38]. The walkability index was adapted from the walkability index developed by Frank et al. [39] and calculated using following formula: Walkability = (2*z-connectivity)+(z-residential density)+(z-land use mix) [38]. Only neighborhoods in the top and bottom walkability quartiles were selected, representing high and low walkability, respectively.

#### Self-reported potential moderators: perceived neighborhood physical environment

Perceptions of the neighborhood physical environment were assessed using the Neighborhood Environment Walkability Scale [40]. The following nine perceived environmental factors were included in the present study: land use mix diversity; access to recreational facilities; access to services; connectivity of the street network; physical barriers to walking; infrastructure for walking; aesthetics; safety from crime; and safety from speeding motorized traffic. A description of these variables’ content and scoring is provided in Table 1.

**Table 1:** Content and scoring of the perceived environment variables.

Variable name (number of items)^a^	Content	Scoring
Land use mix diversity (6)	Distance to local facilities (e.g., bakery, post office, sports accommodation)	5-point scale
Access to recreational facilities (2)	Accessibility of local recreational facilities (e.g., park, open space area, swimming pool, sports accommodation)	5-point scale
Access to services (3)	Accessibility of local shops and services (e.g., easy to walk to transit stop, easy walking distance to shops)	4-point Likert scale
Connectivity (2)	Connectedness of the street network (e.g., presence of intersections, possibility for alternative routes)	4-point Likert scale
Physical barriers to walking (2)	Presence of barriers that make it difficult to directly walk to places (e.g., freeways, canals, dead-end-streets)	4-point Likert scale
Infrastructure for walking (3)	Presence and quality of walking infrastructure (e.g., presence of sidewalks, streets lit at night, crosswalks)	4-point Likert scale
Aesthetics (4)	Presence of aesthetic features (e.g., trees along the streets, attractive buildings, natural sights)	4-point Likert scale
Safety from crime (3)	Perceived safety from crime-related features (e.g.; safety for walking outdoors during the day/in the evening, low crime rate)	4-point Likert scale
Safety from motorized traffic speeding (2)	Perceived low speed of motorized traffic in the street (e.g., cars driving slow, low speed limit)	4-point Likert scale

^a^All variables were calculated by averaging the scores on the items included

#### Outcome measures: self-reported walking and objective MVPA

Self-reported PA levels were assessed using the long International Physical Activity Questionnaire (IPAQ; www.ipaq.ki.se, last 7 days interview version). In a previous study among Belgian older adults [41] acceptable test-retest reliability of IPAQ was found. Within a time frame of the last seven days, all participants reported the number of days and the average time on such a day (hours and minutes) they spent doing work-related, domestic, transport-related and recreational PA. Participants were asked to report only those activities with a minimum duration of 10 consecutive minutes. Walking is the most popular type of older adults’ PA and can be considered a cheap, accessible activity (even for those with lower levels of physical functioning) that can be easily integrated into the older adults’ daily routine [42,43]. Moreover, since the present study examined moderating effects of the physical environment, indoor activities (e.g., household chores) or activities that are executed in private settings (gardening, (voluntary-) work-related physical activity), are less likely to be affected by the neighborhood physical environment and were therefore not taken up in the analyses. Therefore, only self-reported weekly minutes of ‘walking for transport’ and ‘walking for recreation’ were used as dependent variables in the present study. Data on total self-reported PA (calculated as a summary score of all reported domain-specific PA, expressed in minutes per week) were used for preliminary screening on outliers (i.e., self-reports of ≥ 6720 minutes/week or 16 hours/day were excluded [44]). Based on this preliminary screening, 21 participants were excluded from the analyses.

Because walking alone does not capture all types of older adults’ weekly PA, a measure of overall weekly MVPA was included in the analyses as well. As older adults are likely to over-report their overall MVPA levels [41], MVPA was included in the study as an objectively-assessed outcome measure. MVPA was objectively assessed with ActiGraph GT3X(+) accelerometers (ActiGraph, Fort Walton Beach, FL, USA), which are valid and reliable tools to measure PA levels, also in older adults [45–47]. Accelerometers were attached to an adjustable elastic waist belt and worn above the right hip bone for seven consecutive days. Data capturing the vertical plane were collected using 60-second epochs, according to the recommendations for accelerometer use in older adults [48]. Raw accelerometer data were downloaded and exported to CSV files with the Actilife 6.0 software (Actigraph, Fort Walton Beach, FL, USA), which were subsequently screened, cleaned and scored using MeterPlus 4.3 (Santech, Inc.). A valid day was defined as a minimum of 10 wearing hours and participants with less than five valid days of data were excluded for analysis. Periods covering ≥ 90 minutes of consecutive zeros were categorized as ‘non-wearing’ [49]. MVPA was defined as registrations ≥ 1,952 counts.min^-1^ [50], which were subsequently converted into weekly minutes of MVPA. Twenty-eight participants were excluded for analysis because of accelerometer failure, and 21 were excluded because they had fewer than five valid accelerometer wearing days [48].

### Statistical analyses

The analytical sample of the present study consisted of 438 older adults. Descriptive statistics were calculated using SPSS 22.0 (SPPS Inc., Chicago, IL, USA). Because PA variables were positively skewed, they were square root transformed to improve normality in the data. Except for descriptive statistics, which were calculated with the raw data, square root transformed variables were used in all analyses reported below. Possible multicollinearity of the environmental predictors was checked by calculating Pearson correlations. If correlation coefficients were higher than 0.60, the predictor showing the lowest correlation with the outcome measure was excluded for analysis. Multicollinearity only occurred between land use mix diversity and access to services (r = 0.61). Land use mix diversity was retained for the analyses with transport walking and MVPA as outcome measures, whereas access to services was retained in the analyses for recreational walking. Next, multilevel linear regression models (two level: neighborhood-participant) were conducted in MLwiN 2.30 to determine the main effect of physical functioning on each of the three PA outcome measures and to assess moderating effects of environmental factors on this association. Objective neighborhood walkability was included as a categorical factor (high vs. low). All perceived environmental variables were entered as continuous variables and centered around their mean. Analyses were conducted in two consecutive steps. In a first step (Step1), for each PA measure, moderating and main effects were calculated separately for each environmental variable, by entering in the model the respective covariates, main terms for physical functioning and the environmental variable, and an interaction term between functioning and the environmental variable (for these interim results, see S1 and S3 Tables). In a second step (Step2), a multivariable model was built, including all main and interaction terms yielding p<0.10 in the first step. For these multivariable models, statistical significance was set at p<0.05 for interpreting main effects and at p<0.10 for interpreting moderating effects as interactions have lower power [51]. For both of the walking outcome measures (i.e. transport and recreation), all analyses were adjusted for gender, educational attainment, living situation, age and neighborhood income, since these variables were significantly related to at least one of the walking variables. Analyses using objectively-measured MVPA as the outcome measure were also adjusted for the number of valid wearing days and the number of valid wearing hours per day. Furthermore, as in previous analyses [30], walkability and MVPA were only associated in low-income neighborhoods, three-way interactions between the environmental variables, neighborhood income and physical functioning were examined for this outcome measure. To visualize moderating effects, the predicted PA measure (transformed minutes/week values) was plotted against physical functioning in interaction with the environmental variable.

## Results

### Sample characteristics

Sample demographics are displayed in Table 2. Furthermore, Table 2 shows that physical functioning was high on average with a mean physical functioning score of 9 ± 2 out of 12. Regarding PA, participants self-reported an average of 86 ± 141 weekly minutes of transport walking (median 30 min/wk; IQR 0–120 min/wk); 83 ± 159 weekly minutes of recreational walking (median 0 min/wk; IQR 0–120 min/wk); and accelerometer data showed that on average 111 ± 117 weekly minutes were spent doing moderate-to-vigorous PA (median 70 min/wk; IQR 23–162 min/wk).

**Table 2:** Sample characteristics.

	N = 438
**Socio-demographics**	
Gender (% female)	54.1
Age in years	74.3 ± 6.2
Living situation (% with partner)	65.8
Educational level (% tertiary)	38.4
Former occupation (%)	
*household*	18.2
*blue collar*	27.1
*white collar*	54.7
**Independent variable**	
Physical functioning score (/12)	8.7 ± 2.2
Physical functioning score range (min–max)	2–12
**Potential moderators**	
Land use mix diversity (/5)	3.6 ± 0.9
Access to recreational facilities (/5)	2.8 ± 1.3
Access to services (/4)	3.5 ± 0.7
Physical barriers to walking (/4)	2.9 ± 0.4
Connectivity (/4)	2.8 ± 0.8
Infrastructure for walking (/4)	3.1 ± 0.9
Aesthetics (/4)	2.6 ± 0.7
Safety from crime (/4)	3.3 ± 0.7
Safety from speeding traffic (/4)	2.3 ± 1.0
**Dependent variables**	
Transport walking^a^ (min.wk^-1^) Mean ± SD	86.1 ± 140.9
*Median; IQR*	30.0; 0.0–120.0
Recreational walking^a^ (min.wk^-1^) Mean ± SD	83.0 ± 159.1
*Median; IQR*	0.0; 0.0–120.0
MVPA^b^ (min.wk^-1^) Mean ± SD	110.5 ± 116.8
*Median; IQR*	70.0; 23.0–162.0

Numbers represent mean ± standard deviations, unless indicated otherwise. PA = physical activity; MVPA = moderate-to-vigorous PA; SD = standard deviation; IQR = interquartile range.

^a^ self-reported

^b^ accelerometer-based

### Self-reported walking for transport

**Fig 1:**
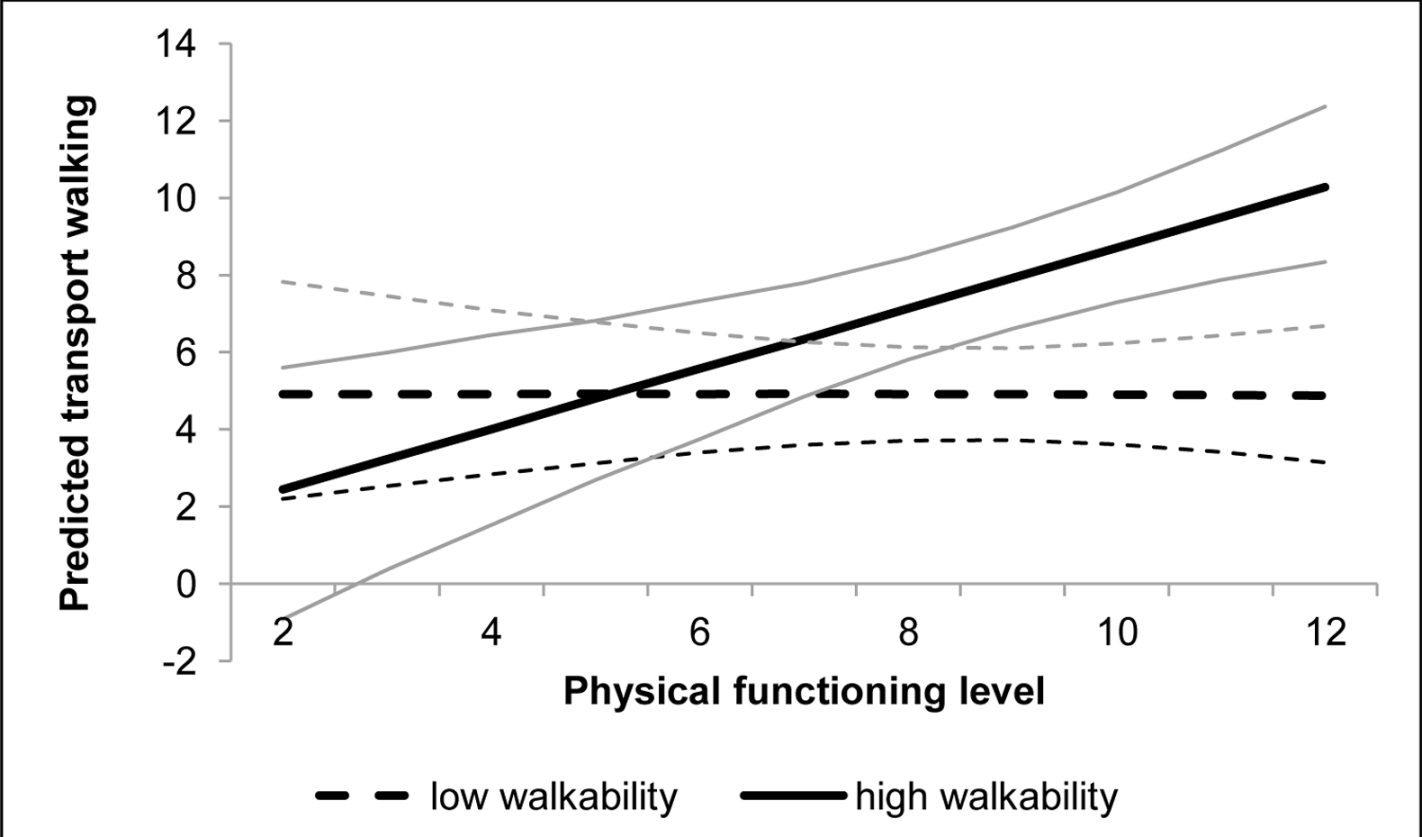
Interaction between physical functioning and neighborhood walkability for the predicted transport walking. Plot represents the predicted transport walking of the square root transformed variable for high-walkability (thicker full line) and low-walkability (thicker dashed line) neighborhood residents, and their confidence intervals (thinner full and thinner dashed lines).

As shown in Table 3, multivariable analyses indicated that objective neighborhood walkability moderated the association between older adults’ physical functioning and weekly minutes of transport walking (B = 0.792; p = 0.003). This moderating effect is plotted in Fig 1 and shows that only for older adults living in high-walkable neighborhoods, physical functioning was positively associated with more transport walking (p<0.001). In low-walkable neighborhoods, there was no difference in transport walking levels depending on the lower-extremity function of older adults (p = 0.964). None of the perceived environmental factors moderated the association between physical functioning and older adults’ transport walking.

**Table 3:** Multivariable model for transport walking.

	**Main effect functioning**		
	**B ± SE**		
	**-0.009 ± 0.197**		
		**Main effect environmental factor**	**Functioning x environment**
		**B ± SE**	**B ± SE**
Walkability (ref = low)		2.797 ± 0.959*	0.792 ± 0.271*
LUM diversity		0.283 ± 0.457	
Access recr. facilities		0.370 ± 0.295	
Connectivity		1.401 ± 0.412*	
Safety crime		-1.339 ± 0.457*	-0.326 ± 0.201

B = regression coefficient; SE = standard error

* p<0.05

The transport walking variable was square root transformed; Inclusion of the environmental predictors in the multivariable model were based on results of separate analyses for each environmental predictor (see S1 Table). The multivariable model was adjusted for gender, age, living situation, educational attainment, and neighborhood income.

### Self-reported walking for recreation

None of the environmental factors moderated the positive association between older adults’ physical functioning levels and their self-reported walking for recreation in the separate models (Step 1; see S1 Table). Consequently, no moderating effects were examined in the multivariable analyses (Step 2; see S2 Table for results on main effects only).

### Accelerometer-based MVPA

**Fig 2:**
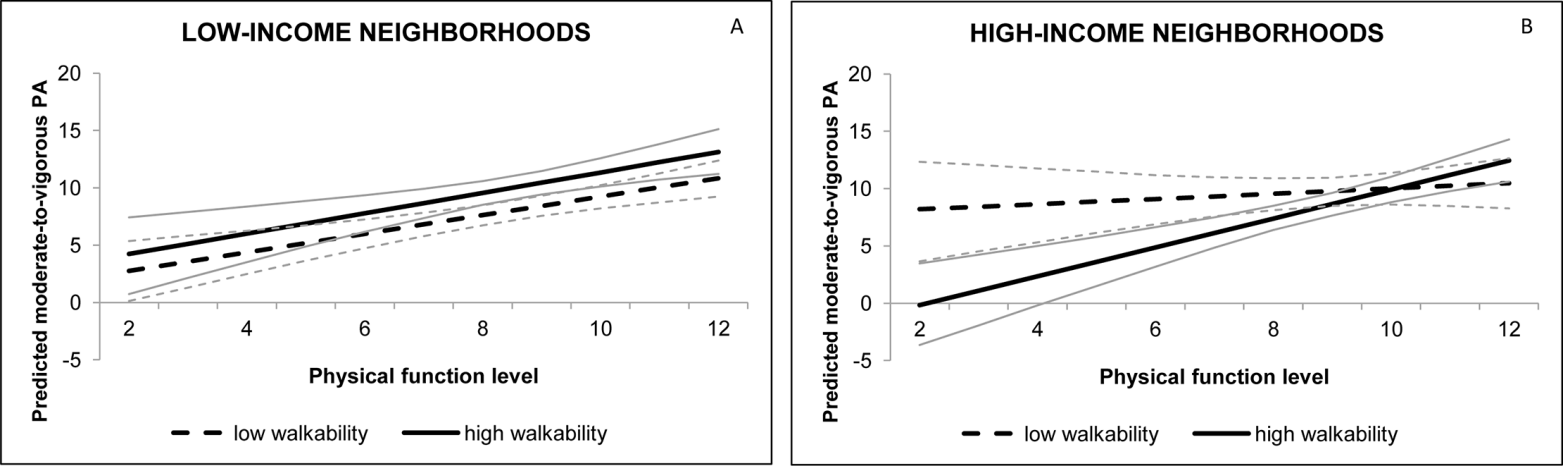
Three-way interaction between physical functioning, neighborhood income, and neighborhood walkability for the predicted MVPA. Fig 2A. shows the moderating effect of neighborhood walkability on the functioning-MVPA association in low-income neighborhoods Fig 2B. shows the moderating effect of neighborhood walkability on the functioning-MVPA association in high-income neighborhoods Plots represent the predicted MVPA of the square root transformed variable for high-walkability (thicker full lines) and low-walkability (thicker dashed lines) neighborhood residents, and their confidence intervals (thinner lines).

Results for the accelerometer-based MVPA outcome measure are reported in Table 4. The multivariable model indicated a three-way interaction effect between neighborhood income, neighborhood walkability and older adults’ physical functioning. This interaction is depicted in Fig 2. As can be seen from the plots, neighborhood walkability did not moderate the association between physical functioning and MVPA in low-income neighborhoods (p = 0.769; Fig 2A.), as both in high- and low-walkable neighborhoods, there was an overall strong positive association (p<0.001) between physical functioning and accelerometer-based MVPA. In contrast, in high-income neighborhoods (Fig 2B.), walkability moderated the association between physical functioning and MVPA: only in the high-income, high-walkable neighborhoods, higher physical functioning scores were associated with higher MVPA levels (p<0.001), whereas no significant association was observed between physical functioning and MVPA in high-income, low-walkable neighborhoods (p = 0.422). Neither three-way income x functioning x environment, nor two-way functioning x environment interactions were observed for the moderating analyses with the perceived physical environmental variables.

**Table 4:** Multivariable model for accelerometer-derived MVPA.

	**Main effect**				
	**B ± SE**				
Functioning	0.803 ± 0.190**				
Neighborhood (ref = low)	1.497 ± 0.786^¥^				
Functioning x income	-0.565 ± 0.337^¥^				
**Environmental predictors**		**Main effect predictor**	**Income x predictor**	**Functioning x predictor**	**Functioning x income x predictor**
		**B ± SE**	**B ± SE**	**B ± SE**	**B ± SE**
Walkability (ref = low)		1.995 ± 0.707**	-3.385 ± 1.167*	0.088 ± 0.301	0.927 ± 0.523^¥^
Land use mix diversity		1.017 ± 0.470^¥^	-1.389 ± 0.627*		
Connectivity		0.056 ± 0.296		0.175 ± 0.143	
Walking infrastructure		0.335 ± 0.400	0.859 ± 0.616	-0.136 ± 0.181	0.097 ± 0.289

B = regression coefficient; SE = standard error. The MVPA variable was square root transformed; Inclusion of the environmental predictors in the multivariable model were based on results of separate analyses for each environmental predictor (S3 Table). The multivariable model was adjusted for number of accelerometer wearing days, number of accelerometer wearing hours per valid day, gender, age, living situation, and educational attainment.

** p<0.001

* p<0.05

^¥^ p<0.10

## Discussion

The present study examined the possible moderating role of the neighborhood physical environment on the association between physical functioning and PA in community-dwelling Belgian older adults. Outcome measures used in this study were transport walking, recreational walking and objectively-measured MVPA.

Regarding the transport walking outcome, a significant interaction was observed between neighborhood walkability and physical functioning. In low-walkable neighborhoods, transport walking levels were generally low for both older adults with poorer and better functioning levels, whereas for older adults living in high-walkable neighborhoods, better physical functioning was associated with higher levels of transport walking. A previous study on the same sample already described a main effect of neighborhood walkability on transport walking in these Belgian older adults [30]. Because in the present study, the highest levels of transport walking were observed for high-walkability neighborhood residents with the highest functioning levels, our results now add to this knowledge by suggesting that walkability may be most important for those older adults who still have a relatively good lower-extremity physical functioning. Although our findings are not in line with the present study’s hypothesis and press-competence theory [31] (i.e. it was expected that the functioning-PA association would be more pronounced in low-walkable neighborhoods), the direction of interaction confirms results from previous US studies examining functioning x environment interactions in older adults [29,35].

The observed synergistic effects between better functioning and living in a high-walkable neighborhood on active transport could suggest that, when city planners and health promoters are aiming to keep older adults living independently (i.e., functionally fit) for as long as possible, a high-walkable residential neighborhood may be a facilitating factor to keep the functionally fit physically active. Our results could indicate that when there might be no easy access to relevant destinations in the neighborhood (i.e., low walkability), this functioning benefit regarding transport walking may be neutralized. A longitudinal study among US older adults showed that the odds for over-time declines in transport walking was lower if older adults lived in a high-walkable environment [52]. Our own study findings might imply that age-related declines in PA could also be related to functional declines, which may occur less rigorously when older adults are living in an activity-supportive neighborhood. However, longitudinal assessments of over-time changes in transport walking and on the interplay between walkability and functioning are required to confirm this suggestion and to provide insight into direction of causality.

Despite above-mentioned synergistic effects, the plot for transport walking seemed not to confirm that high vs. low walkability was associated with higher PA levels for older adults with lower physical functioning levels. This may indicate that the objective physical environment could be less important for community-dwelling older adults with poorer physical functioning levels, and/or that other factors (e.g. fear of falling [16] or declining cognition [53]) might be more essential in predicting PA in this population subgroup. Nevertheless, our results are similar to the findings of two previous studies conducted among US older adults [29,35], who also observed positive associations between physical functioning and older adults’ PA when the objectively-measured physical environment was more activity-friendly. Also concordant with this US literature is the inconclusive role of environmental *perceptions* as moderators on the functioning-PA association in older adults. For instance, perceptions of connectivity and land use mix diversity, two walkability-related components, were not identified as moderators on the functioning-transport walking association in the present study, whereas *objective* neighborhood walkability was. However, the observed lack of moderation by environmental perceptions is not necessarily negative for future intervention development, as it may imply that functioning is equally related to older adults’ PA in those with better and with poorer environmental perceptions. So, older adults with higher functioning levels, but poorer perceptions, might still benefit from living in an objectively-determined high-walkable neighborhood despite these poor perceptions.

Next to the observed interaction effect on transport walking, a three-way interaction between neighborhood income, neighborhood walkability and physical functioning was observed in the analyses for objectively-measured MVPA. The interaction between functioning and walkability showed that the functioning-MVPA association was significant only in high-walkable, high-income neighborhoods. In these high-income, high-walkable neighborhoods, those older adults with the highest functioning levels, also accumulated the highest number of weekly MVPA minutes. It is difficult to provide a univocal explanation for this three-way interaction. Regarding our findings on transport walking, it seemed that older adults with higher functioning levels needed to live in high-walkable neighborhoods to benefit from their better physical functioning. For MVPA, findings suggest that the benefit of good physical functioning does not only depend on a high walkability, but also on generally higher income levels in the neighborhood. The mechanism behind this observation, however, cannot be revealed from the present study findings. It could be possible that the high-income, high-walkable neighborhoods have more destinations appropriate for the elderly (e.g., nicer shops) or a better social capital and higher PA modeling levels for this population subgroup, which makes it easier to engage in MVPA in these neighborhoods. Future studies, however, are required to investigate these possible mechanisms.

The present study results indicated that none of the environmental variables moderated the association between physical functioning and recreational walking, suggesting an equal importance of physical functioning to explain levels of recreational walking for those living in an activity-friendly versus non-friendly environment. However, more plausibly, this lack of moderation implies a generally lower importance of the neighborhood physical environment for recreational walking. Although some studies observed positive associations between the physical environment and recreational activities in older adults [25,54,55], the explanatory role of the physical environment for recreational PA in older adults is still inconclusive [34]. Also a previous study within the BEPAS Seniors sample has already shown a lack of association between neighborhood walkability and recreational walking and a higher importance of psychosocial factors to explain this type of walking [30]. Thus, older adults’ recreational activities may be affected to a lesser extent by the environment itself, because physical functioning needs to be at a certain basic level to still engage in (and possibly enjoy) recreational walking. Furthermore, it may be possible that other factors, such as fear of falling, are more important to explain the association between functioning and recreational walking, but it was beyond the scope of the present study to investigate the role of falling-related fear.

### Strengths and limitations

A first strength of the present study is that older adults’ physical functioning was measured objectively by the Short Physical Performance Battery (SPPB) [37]. Although SPPB only assesses lower-extremity functioning, the SPPB outcome measure can be considered a good proxy for overall walking-related functioning because walking may not necessarily require better upper-body functioning. Secondly, self-reported walking levels were assessed through face-to-face interviews. In adults, it was found that over-reporting bias was smaller when the IPAQ interviewer-administered version versus the self-administered version was used [56]. Guidance by the interviewer can be even more beneficial in older adults, because this age group may experience more cognitive difficulties when responding to a questionnaire [57]. A third strength of the present study is that older adults’ MVPA levels were assessed objectively, because overall MVPA levels are likely to be over-reported by older adults [41]. Fourthly, these results apply to a specific Western-European geographical setting, in which the physical environment may differ substantially from other regions. For instance, low-walkable neighborhoods in Belgium may be categorized as high-walkable in the US [58], because in Belgium, differences between low and high walkability are generally smaller than those in the US. Nonetheless, given that results of this Belgian study confirmed those from the US is promising, because even in regions with smaller differences between low and high walkability, similar associations were found. Limitations include that the present study had a cross-sectional design and causality of the findings could not be established. As functional fitness generally decreases with increasing age [2,59], longitudinal studies are recommended to examine possible changes in the observed relationships over time. Additionally, the development of interventions to increase older adults’ PA levels or the use of natural experiments are needed to identify causality of the present study findings. Secondly, generalizability of the present study findings to other geographic areas should be cautiously interpreted. For example, despite the observed similarities between the present study findings and those observed in the US mentioned above, regions such as Hong Kong are characterized by ultra-dense urban designs and more research needs to be conducted to see whether the findings also apply to those geographical regions. Moreover, socio-cultural differences might play a role and therefore, even in countries with similar geographical characteristics, more research is needed to confirm our findings. Thirdly, a higher percentage of older adults were higher educated than the Belgian population [60] and also a higher percentage of older adults lived with a partner. Given that higher educational attainment and having a partner have both been associated with higher levels of PA, the observed findings with regard to the functioning-PA relation could slightly differ from those that would be observed in the general Belgian population and generalization to the lower-educated and those living alone may be jeopardized. For instance, less functionally fit older adults who do not live with a partner might have to rely on themselves for all daily tasks, and might therefore engage in more PA than older adults who can rely on a partner to help them with certain tasks and activities. Otherwise, those living with a partner might be triggered more by their partner to engage in PA, even if their functioning levels are poorer, whereas older adults without a partner might not have as much social support or modeling by a significant other, and may therefore engage in less PA. Taking this into account, the research questions posed in the present study need further investigation, and should be tested in samples with a lower proportion of highly-educated older adults, and with a higher proportion of older adults living alone. Besides, only community-dwelling older adults were included in the study. Because of this and taking the other inclusion criteria into account, mean levels of physical functioning were rather high. If mean functioning levels would have been lower, a greater difference in PA levels could have been expected between those with the poorest versus the best functioning levels. Moreover, no interactions between functioning and perceived environmental factors may have been observed in the current study, because some of the perceived environmental factors may be less important to older adults with higher levels of physical functioning. Perhaps, significant interactions between functioning and some of the perceived environmental factors could be present if a broader spectrum of functioning would be represented in the sample.

## Conclusions

According to the present study findings, objective neighborhood walkability moderated the association between physical functioning and transport walking in older adults, and the association between physical functioning and objective MVPA, however, the latter only in high-income neighborhoods. Our findings might demonstrate that interventions aimed at increasing transport-related walking and high-income neighborhood MVPA of the functionally fitter older adults could focus on enhancing walkability through the provision of local shops and services, or recreational areas with high accessibility. Hence, high neighborhood walkability might thus be one of the factors contributing to primary prevention of declining functioning and decreasing PA levels in community-dwelling older adults. In contrast to the objective walkability, results suggest that the perceived physical environment has less influence on the functioning-PA association in Belgian older adults. Moreover, none of the environmental variables (neither objective, nor perceived) moderated the association between functioning and recreational walking, suggesting that functioning itself is a strong predictor of recreational walking, or that other factors, apart from the objective physical environment, moderate the functioning-recreational walking association.

The present study was conducted in a sample of community-dwelling Belgian older adults, with higher levels of educational attainment. It is recommended that the research questions posed in the present study are replicated in studies among older adults living in other geographical settings and with a broader variance in educational and functional level. Moreover, longitudinal studies, investigating over-time changes in the observed associations, are highly encouraged.

## Supporting Information

S1 Table Main and two-way interaction effects on self-reported transport walking and recreational walking (first step of the analyses). (PDF)

S2 Table Multivariable model for recreational walking. (PDF)

S3 Table Main, two- and three-way interaction effects on objectively-measured MVPA (first step of the analyses). (PDF)
