# Supplementary material for: The Association between Belgian Older Adults’ Physical Functioning and Physical Activity: What Is the Moderating Role of the Physical Environment?
Source: PLoS One. 2016 Feb 12;11(2):e0148398. doi: 10.1371/journal.pone.0148398 (PMC4752465; doi:10.1371/journal.pone.0148398)
Supplement: S1 Table — (PDF) [file pone.0148398.s001.pdf]

S1 Table. Main and two-way interaction effects on self-reported transport walking and recreational walking (first step of the analyses)

	Transport walking			Recreational walking		
	**Main effect function**	**Main effect env. factor**	**Interaction function x env.**	**Main effect function**	**Main effect env. factor**	**Interaction function x env.**
	**B ± SE**	**B ± SE**	**B ± SE**	**B ± SE**	**B ± SE**	**B ± SE**
Walkability (ref= low)	-0.033 ± 0.201	**4.730 ± 0.991****	**0.935 ± 0.274****	**0.634 ± 0.194***	-0.405 ± 0.741	0.448 ± 0.332
LUM diversity	**0.304 ± 0.167[¥]**	**1.290 ± 0.455***	0.244 ± 0.152	[a]	[a]	[a]
Access recr. facilities	**0.311 ± 0.166[¥]**	**0.915 ± 0.302****	0.108 ± 0.107	**0.601 ± 0.196***	0.318 ± 0.285	0.086 ± 0.127
Access to services	[a]	[a]	[a]	**0.605 ± 0.195***	0.676 ± 0.530	0.215 ± 0.236
Connectivity	**0.408 ± 0.162***	**1.756 ± 0.412****	0.173 ± 0.176	**0.622 ± 0.193***	**0.958 ± 0.447***	0.185 ± 0.214
Barriers walking	**0.395 ± 0.165***	-0.371 ± 0.705	0.101 ± 0.316	**0.636 ± 0.194****	-0.731 ± 0.828	0.122 ± 0.379
Walking infrastructure	**0.377 ± 0.166***	0.445 ± 0.472	0.200 ± 0.153	**0.597 ± 0.194***	**0.723 ± 0.397[¥]**	0.061 ± 0.181
Aesthetics	**0.394 ± 0.165***	0.366 ± 0.527	0.303 ± 0.202	**0.642 ± 0.193****	**0.926 ± 0.553[¥]**	0.177 ± 0.235
Safety crime	**0.414 ± 0.163***	**-1.322 ± 0.478****	**-0.436 ± 0.204***	**0.638 ± 0.194****	-0.054 ± 0.554	-0.102 ± 0.248
Safety traffic speed.	**0.361 ± 0.168***	0.330 ± 0.325	-0.129 ± 0.140	**0.639 ± 0.196****	-0.090 ± 0.376	0.037 ± 0.170

** p<0.001; * p<0.05; ¥ p<0.10

Walking variables were square root transformed; Main and moderating effects were calculated for each environmental variable separately, adj. for gender, age, living situation, education, and neighborhood income

[a]Because of multicollinearity (r>0.60) between land use mix diversity and access to services, this predictor was excluded from the analyses; For each of both PA outcome measures, the main and interaction terms in bold font (p<0.10) were simultaneously included in a multivariable model (see Results, Table 3 for the multivariable results regarding transport walking; see Additional file S2 for the multivariable results regarding recreational walking).
