# Supplementary material for: The Association between Belgian Older Adults’ Physical Functioning and Physical Activity: What Is the Moderating Role of the Physical Environment?
Source: PLoS One. 2016 Feb 12;11(2):e0148398. doi: 10.1371/journal.pone.0148398 (PMC4752465; doi:10.1371/journal.pone.0148398)
Supplement: S2 Table — (PDF) [file pone.0148398.s002.pdf]

S2 Table. Multivariable model for recreational walking

	Main effects
	**B ± SE**
Functioning	0.597 ± 0.193*
Connectivity	0.675± 0.477
Walking infrastructure	0.581± 0.422
Aesthetics	0.935± 0.555¥

* p<0.05; ¥ p<0.10

Walking variable was square root transformed; This model was adjusted for gender, age, living situation, education, and neighborhood income. Because for recreational walking, no functioning x environmental factor interactions were identified in Step 1 of the analyses (single models), only main effects of functioning and environmental factors are reported in this table.
