# Supplementary material for: The Association between Belgian Older Adults’ Physical Functioning and Physical Activity: What Is the Moderating Role of the Physical Environment?
Source: PLoS One. 2016 Feb 12;11(2):e0148398. doi: 10.1371/journal.pone.0148398 (PMC4752465; doi:10.1371/journal.pone.0148398)
Supplement: S3 Table — (PDF) [file pone.0148398.s003.pdf]

S3 Table. Main, two- and three-way interaction effects on objectively-measured MVPA (first step of the analyses)

Environmental factor	Main effect functioning	Main effect income (ref=low)	Main effect env. factor	Income x functioning	Income x env. factor	Functioning x env. factor	Functioning x income x env. factor
	B ± SE	B ± SE	B ± SE	B ± SE	B ± SE	B ± SE	B ± SE
Walkability (ref=low)	**2.789 ± 0.712****	0.756 ± 0.706	**0.861 ± 0.187****	-0.526 ± 0.266	**-2.823 ± 1.013****	0.151 ± 0.276	**0.793 ± 0.391***
LUM diversity	**0.803 ± 0.122****	-0.201 ± 0.660	**1.382 ± 0.449***	-0.044 ± 0.199	**-1.174 ± 0.575***	0.023 ± 0.110	0.198 ± 0.228
Access recr. facilities	**0.852 ± 0.122****	-0.511 ± 0.704	0.326 ± 0.204	-0.061 ± 0.200	-0.278 ± 0.391	0.049 ± 0.077	0.101 ± 0.162
Connectivity	**0.889 ± 0.120****	-0.511 ± 0.733	0.289 ± 0.294	-0.039 ± 0.200	-0.758 ± 0.588	**0.257 ± 0.128***	0.160 ± 0.255
Barriers walking	**0.888 ± 0.121****	-0.603 ± 0.755	0.181 ± 0.504	-0.048 ± 0.201	-1.367 ± 1.003	-0.208 ± 0.227	0.194 ± 0.457
Walking infrastructure	**0.908 ± 0.155****	-0.349 ± 0.672	**0.969 ± 0.448***	-0.043 ± 0.204	-0.364 ± 0.589	-0.069 ± 0.167	**0.433 ± 0.224[¥]**
Aesthetics	**0.885 ± 0.121****	-0.744 ± 0.742	0.334 ± 0.366	-0.052 ± 0.200	0.308 ± 0.721	0.090 ± 0.145	-0.238 ± 0.299
Safety crime	**0.887 ± 0.121****	-0.614 ± 0.766	0.370 ± 0.341	-0.041 ± 0.200	0.052 ± 0.680	-0.052 ± 0.148	0.086 ± 0.299
Safety traffic speeding	**0.861 ± 0.123****	-0.603 ± 0.732	0.230 ± 0.232	-0.049 ± 0.201	-0.066 ± 0.460	0.047 ± 0.101	0.232 ± 0.203

** p<0.001; * p<0.05; [¥] p<0.10

The outcome variable (MVPA) was square root transformed; main effects, two-way and three-way interactions were calculated for each environmental factor, adj. for number of valid accelerometer wearing days, number of accelerometer hours on valid days, gender, age, living situation and educational attainment. Main and interaction terms in bold font (p<0.10) were simultaneously included in a multivariable model (see Results, Table 4).
